# Changes in Gloss Alteration, Surface Roughness, and Color of Direct Dental Restorative Materials after Professional Dental Prophylaxis

**DOI:** 10.3390/jfb15010008

**Published:** 2023-12-23

**Authors:** Aya Miyashita-Kobayashi, Akiko Haruyama, Keigo Nakamura, Chia-Ying Wu, Akihiro Kuroiwa, Nobuo Yoshinari, Atsushi Kameyama

**Affiliations:** 1 Department of Oral Health Promotion, Graduate School of Oral Medicine, Matsumoto Dental University, Shiojiri, Nagano 399-0781, Japan; aya.kobayashi@mdu.ac.jp (A.M.-K.); kaei.go@mdu.ac.jp (C.-Y.W.); nobuo.yoshinari@mdu.ac.jp (N.Y.); 2 Department of Operative Dentistry, Endodontology and Periodontology, School of Dentistry, Matsumoto Dental University, Shiojiri, Nagano 399-0781, Japan; keigo.nakamura@mdu.ac.jp; 3 Department of Operative Dentistry, Cariology, and Pulp Biology, Tokyo Dental College, Chiyoda-ku, Tokyo 101-0061, Japan; akiharu@tdc.ac.jp; 4 Department of Dental Materials Science, School of Dentistry, Matsumoto Dental University, Shiojiri, Nagano 399-0781, Japan; akihiro.kuroiwa@mdu.ac.jp

**Keywords:** resin-based composite, glass–ionomer cement, surface gloss, surface roughness, color change, 3D measuring laser microscopy

## Abstract

In the context of optimizing dental care for patients who are elderly, the purpose of this in vitro study was to evaluate the surface gloss (with a micro-area gloss meter) of, surface roughness (with a compact surface roughness measuring instrument) of, and color change (with a dental colorimeter) in two commercially available injectable resin-based composites (Estelite Universal Flow (EUF) and Beautifil Flow Plus F00 (BFP)) as well as two glass–ionomer cements (GC Fuji II LC CAPSULE (FLC) and GC Fuji IX GP EXTRA CAPSULE (FGP)), before and after dental prophylaxis. After 24 h, the surfaces of each specimen were polished at 2500 rpm with a prophy brush (Mersage Brush, Shofu) and one-step prophylaxis paste (Prophy Paste Pro, Directa): under 100 or 300 gf load, and for 10 or 30 s, 4× cycles of cleaning. After mechanical cleaning, conditions were found for a significant reduction in the gloss level (EUF, BFP, or FLC; *p* < 0.05) and a significant increase in surface roughness (BFP; 300 gf load, 10 s × four cycles of cleaning). Overall, the longer time or higher prophylaxis load tended to decrease the surface gloss. However, the observed change in surface roughness varied between the restorative materials. There was no color change post-prophylaxis.

## 1. Introduction

In Japan, dental longevity has increased dramatically over the past 30 years. In 1989, the Japanese Ministry of Health (currently the Japanese Ministry of Health, Labour, and Welfare) and the Japan Dental Association set a goal for 80-y-olds to preserve more than 20 teeth per individual (termed the 80/20 movement or 8020 campaign). At the time, only 7% of people who were elderly had achieved that goal [1], yet 51% had done so by 2016 [2,3]. Thus, more people who are elderly are able to chew food satisfactorily, contributing significantly to an improved quality of life [4].

However, the elderly population percentage in Japan is substantially increasing, with those aged ≥65 and ≥80 y accounting for ca. 30% and 10% of the population, respectively. Thus, the need for home dental care is increasing. General health and oral health are strongly associated in the elderly [5,6]; thus, maintaining oral health in the elderly contributes to maintaining general health [7,8,9,10]. For example, professional oral health care by dentists, dental hygienists, and dental nurses in dental clinics as well as home dental care are important in terms of preventing aspiration pneumonia and nutritional disorders [11,12].

Development of root surface caries is often observed in the remaining teeth of patients who are elderly [13]. Early root surface caries can be treated by self-care with aggressive use of high-concentration fluoride toothpaste or fluoride mouth rinse, 5% sodium fluoride varnish, and noninvasive interventions (such as 1.2% acidulated phosphate fluoride or silver diamine fluoride), and can reduce their activity [14,15,16,17]. However, upon severe loss of the tooth structure, restorative procedures with resin-based composites (RBCs) or glass–ionomer cements (GICs) are necessary [18].

Usually, after restoration, the material surfaces are carefully polished by the dentist. This is an important action in terms of maintaining esthetics and preventing plaque stagnation [19,20,21]. There are numerous reports on the impact of polishing methods on the properties of RBC and GIC surfaces [22,23,24,25,26,27,28]. However, there are few reports on how the surface properties are changed by professional oral care [29,30,31,32,33], and it remains a matter of debate. In addition, dental prophylaxis has traditionally involved removing plaque and stains from the tooth surface with a coarse-particle prophylaxis paste, followed by polishing the tooth surface with a fine-particle paste [32]. However, in recent years, many one-step-type prophylaxis pastes have become commercially available. The abrasive particles in one-step prophylaxis pastes are broken into smaller particles with use, resulting in smaller-diameter particles. This might affect the hardness of the polished surface, the load applied during polishing, and the polishing time [33].

The purpose of this study was to investigate the effects of the loading and polishing time of dental prophylaxis with one-step prophylaxis paste on the surface gloss, roughness, as well as color change (regarding RBCs and GICs). Therefore, the null hypotheses of this study are (1) dental prophylaxis to direct dental restorative material does not affect the surface gloss, surface roughness, or color change, and (2) duration and load of dental prophylaxis do not affect the surface gloss, surface roughness, or color change.

## 2. Materials and Methods

### 2.1. Materials

Table 1 shows the materials used in this study. Two types of flowable RBC restorative materials (Estelite Universal Flow (Super Low) and Beautifil Flow Plus F00) and two types of GIC-based restorative materials (GC Fuji II LC CAPSULE and GC Fuji IX GP EXTRA) were used. Prophy Paste PRO was used as the prophylaxis paste. A light-emitting diode (LED; Pencure, J Morita, Tokyo, Japan) was used for light curing; the light intensity was controlled with a hand-held dental radiometer (Bluephase Meter II; Ivoclar Vivadent, Schaan, Liechtenstein) to ensure that the light output was at least 1100 mW/cm^2^ [34].

### 2.2. Specimen Preparation and Group Setting

**Figure 1:**
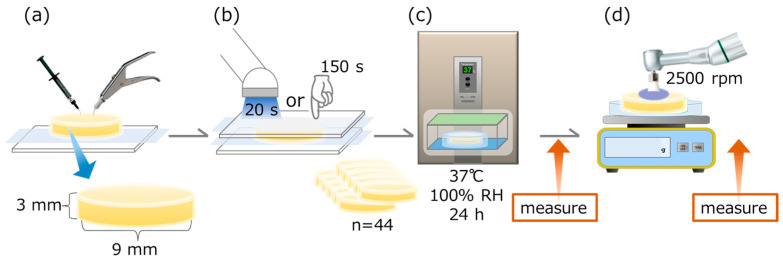
Schematic of specimen preparation. (**a**) Filling materials into the acrylic ring. (**b**) For EUF, BFP, and FLC, light curing from both top and bottom sides for 20 s each. For FGP, finger pressure was kept for 150 s (*n* = 44). (**c**) After storage for 24 h at 37 °C, 100% relative humidity (RH), surface gloss, roughness, and color were measured. (**d**) After prophylaxis, the specimen surface at 2500 rpm, surface gloss, roughness, and color were measured again.

The study design is schematically explained in Figure 1. 

For Estelite Universal Flow and Beautifil Flow Plus, a transparent celluloid strip (GC Celluloid Strips, GC, Tokyo, Japan) on a micro glass slide plate (Matsunami Glass, Kishiwada, Japan) and an acrylic ring (AZ Science, Matsumoto, Japan; outer diameter of 11 mm, inner diameter of 9 mm, and height of 3 mm) was placed on top. The inner surface of the ring was filled with each material and covered with another transparent celluloid strip. The glass slide plate was then irradiated with an LED light-curing device from both sides for 20 s each (*n* = 44 each).

For the Fuji II LC CAPSULE, a transparent celluloid strip was placed on a micro glass slide plate as in the two RBCs, and an acrylic ring (outer diameter of 11 mm, inner diameter of 9 mm, and height of 3 mm) was placed on top. After mixing the GICs in the capsule with a Mixer CM-II (GC), the inner surface of the capsule was filled with each material, covered with another transparent celluloid strip, and then pressed by hand with another micro glass slide plate. The glass slide plate was then irradiated from both sides for 20 s each with an LED light-curing device (*n* = 44).

For Fuji IX GP EXTRA, as in the Fuji II LC CAPSULE, the acrylic ring was filled with an automatically mixed GIC and covered with another transparent celluloid strip, and then a second micro glass slide plate was inserted as well as pressure-applied by hand for 150 s (*n* = 44).

The samples were removed from the acrylic ring and kept in a thermostatic incubator (THS020DA, Advantec, Tokyo, Japan) at 37 °C and 100% relative humidity for 24 h. After 24 h, the samples were removed from the high-temperature incubator and allowed to dry naturally for 30 min in a room-temperature environment. Samples were randomly divided into four groups (*n* = 11) for the following prophylaxis procedures [33]:

Group 1: Load of 100 gf, 10 s, 4×
Group 2: Load of 100 gf, 30 s, 4×
Group 3: Load of 300 gf, 10 s, 4×
Group 4: Load of 300 gf, 30 s, 4×

Ten samples from each group were used to measure the surface gloss, surface roughness, and color, and the remaining sample was used for observation with a 3D measuring laser microscope.

### 2.3. Surface Gloss (Gs)

Measurements were performed on three items before and after professional dental prophylaxis. Surface gloss was measured at a specular angle of 60° with a precision glossmeter (GD-26, Murakami Color Research Laboratory, Tokyo, Japan) with the light source and detector both set at 60° to normal. Before measurement, the glossmeter was calibrated to a standard gloss board (Gs (60°) = 91.9%). Measurements were performed at five sites near the center of each sample to calculate the mean Gs value. The area of each measurement was 5mm in diameter.

### 2.4. Surface Roughness

Surface roughness was measured with a surface profilometer (Surfcom 130A, Tokyo Seimitsu, Tokyo, Japan); the standard cutoff was 0.8 mm, the transverse length was 3.0 mm, and the stylus speed was 0.3 mm/s. Measurements were performed at three sites near the center to calculate mean surface roughness. The three roughness parameters were assessed as follows: Ra (average distance from the profile to the mean line over the length of assessment), Rz (peak-to-valley values of five equal lengths within the profile), and Ry (distance between peak and valley points of the profile, which can be used as an indicator of the maximum defect height within the assessed profile). Each parameter was characterized in accordance with the International Standards Organization (ISO) and Japanese Industrial Standards (JIS).

### 2.5. Initial Color

The initial color of each specimen was measured with a dental spectrophotometer (Vita Easyshade V, Vita Zahnfabrik, Bad Sackingen, Germany), by the CIE L*a*b* color system, where the L* axis indicates the value (lightness/darkness), the a* axis indicates the redness (+a*) or greenness (−a*), and the b* axis indicates the yellowness (+b*) or the blueness (−b*) (L*_Before_, a*_Before_, b*_Before_). Color was measured at one central spot for each specimen under the white background, and the average values of *n* = 10 were used as the color values of each group.

### 2.6. Professional Dental Prophylaxis

Each sample immobilized on a disposable petri dish was placed on a platform on kitchen scales, and prophylaxis paste (0.05 mL) was applied to the center. Prophylaxis was performed with a mounting Merssage Brush No. 2 (Shofu, Kyoto, Japan) on a 16:1 contra-angle slow-speed handpiece (CrossPro, Anthogyr SA, Sallanches, France) connected to a mobile, portable dental unit (Portacube, J. Morita, Tokyo, Japan). After each prophylaxis procedure, the sample was washed with water and air-dried. Afterward, the surface gloss, roughness, and color were measured again.

The data were evaluated statistically by a three-way analysis of variance, followed by post-hoc Tukey’s multiple comparisons at *p* < 0.05 with IBM SPSS Statistics 18 for Windows (IBM, Armonk, NY, USA).

### 2.7. Color Change

The differences between the L*, a*, and b* values (before and after prophylaxis) were calculated as follows:

ΔL* = L*_After_ − L*_Before_  Δa* = a*_After_ − a*_Before_   Δb* = b*_After_ − b*_Before._ (1)

The color change (ΔE*_ab_) was calculated with the following equation:

ΔE*_ab_ = [(ΔL*)^2^ + (Δa*)^2^ + (Δb*)^2^]^1/2^. (2)

### 2.8. Surface Observation with a 3D Measuring Laser Microscope

The center of the sample before and after prophylaxis of each group was observed with a 3D measuring laser microscope (LEXT OLS4000, Olympus, Tokyo, Japan). All measurements were made at a magnification of 50×.

### 2.9. Scanning Electron Microscopy Observations of the Prophylaxis Paste Particles

Four additional Estelite Universal Flow disc-shaped specimens were prepared. Each sample was subjected to prophylaxis 1× under the conditions from Group 1 to Group 4. Afterward, the used prophylaxis paste was rinsed in a disposable petri dish. The water used was carefully removed, and only the paste particles were collected on carbon tape attached to an aluminum slab with a disposable dental microbrush (Benda Micro, Centrix, Shelton, CT, USA). The particles were Au–Pd-coated with a cooled sputter coater (MSP-20-UM Automatic Magnetron Sputter, Vacuum Device, Mito, Japan). The coated specimens were examined through scanning electron microscopy (SEM; SU6600, Hitachi, Tokyo, Japan) at 15 kV.

## 3. Results

### 3.1. Surface Gloss and Roughness of Baseline

Table 2 shows the values of the surface gloss and roughness of the baseline for the various restorative materials. The two RBCs had significantly higher surface gloss and lower roughness than the two GICs (*p* < 0.05). FLC had higher surface gloss and lower roughness than FGP (*p* < 0.05).

### 3.2. Differences in Surface Gloss (Gs) Measured before and after Prophylaxis

Table 3 shows mean gloss values and standard deviations of the experimental groups tested at baseline as well as after prophylaxis, and the difference between the baseline and after prophylaxis. Although the statistical analysis indicates that the measuring period (before vs. after) significantly influenced the results (*F* = 10.260, *p* = 0.026), the prophylaxis condition (*F* = 0.779, *p* = 0.579) and material (*F* = 0.732, *p* = 0.598) were not statistically significant. The interaction among the three factors was significant (*F* = 6.328, *p* < 0.001).

There was no significant difference regarding gloss values between EUF and BFP at the baseline. Gloss values at the baseline of both FLC and FGP were significantly lower than those of both EUF as well as BFP.

For EUF, BFP, and FLC, there was a statistically significant decrease after prophylaxis (*p* < 0.05). However, neither 100 gf–10 s nor 300 gf–10 s corresponded to a statistically significant decrease between the baseline and after prophylaxis for FGP (*p* < 0.001 and *p* = 0.013, respectively).

### 3.3. Differences in Ra Values Measured before and after Prophylaxis

Table 4 shows mean Ra values and standard deviations of the experimental groups tested at the baseline as well as after prophylaxis, and the mean difference between the baseline and after prophylaxis. None of the three factors corresponded to a significant influence (prophylaxis condition: *F* = 0.557, *p* = 0.656; material: *F* = 8.247, *p* = 0.063; measuring period: *F* = 0.063, *p* = 0.819). The interaction among three factors was significant (*F* = 3.013, *p* = 0.002). 

There was no significant difference of the Ra values between EUF and BFP at the baseline. Ra values at the baseline of both FLC and FGP were significantly higher than those of both EUF as well as BFP. Furthermore, the Ra values at the baseline of FGP were significantly higher than those of FLC.

For EUF and FLC, there was no statistically significant difference after prophylaxis (*p* < 0.05). For BFP, no statistically significant difference was found for Ra between the baseline and prophylaxis in each of 100 gf–10 s, 100 gf–30 s, and 300 gf–10 s. However, the Ra of 300 gf–30 s significantly increased after prophylaxis (*p* = 0.011).

For FGP, Ra values were significantly decreased in each of 100 gf–30s, 300 gf–10 s, and 300 gf–30 s.

### 3.4. Differences in Rz Values Measured before and after Prophylaxis

Table 5 shows mean Rz values and standard deviations of the experimental groups tested at the baseline and after prophylaxis, and the mean difference between the baseline and after prophylaxis. None of the three factors corresponded to a significant influence (prophylaxis condition: *F* = 0.233, *p* = 0.871; material: *F* = 8.024, *p* = 0.065; measuring period: *F* = 0.001, *p* = 0.974). There was a significant interaction among the three factors (*F* = 2.286, *p* = 0.017).

Rz values of FGP at the baseline were significantly higher than those of the other three materials.

For FGP, both 300 gf–10 s and 300 gf–30 s corresponded to a significant decrease in the Rz values after prophylaxis (*p* < 0.001, *p* < 0.001, respectively).

### 3.5. Differences in Ry Values Measured before and after Prophylaxis

Table 6 shows mean Ry values and standard deviations of the experimental groups tested at the baseline as well as after prophylaxis, and the mean difference between the baseline and after prophylaxis. Although the factors material and measuring period did not correspond to a significant influence (material: *F* = 9.100, *p* = 0.059; measuring period: *F* = 0.109, *p* = 0.763), the factor prophylaxis condition did (*F* = 6.545, *p* = 0.012). Although there was a statistically significant interaction between the factors measuring period and material (*F* = 17.994, *p* < 0.001), the interaction among the three factors did not correspond to a significant influence (*F* = 1.210, *p* = 0.288).

Ry values of FGP at the baseline were significantly higher than those of the other three materials. For FGP, both 300 gf–10 s and 300 gf–30 s corresponded to significantly decreased Ry values after prophylaxis (*p* < 0.001, *p* < 0.001, respectively).

### 3.6. Color Change

Table 7 shows mean ΔE*ab values of each experimental group. In all conditions, the color difference was smaller than the threshold value (ΔE*ab > 3.3) at which humans can visually perceive color differences [35,36].

### 3.7. Three-Dimensional Measuring Laser Microscopy

**Figure 2:**
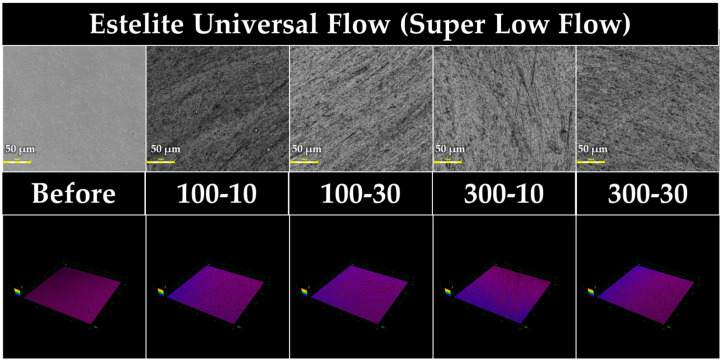
Representative 3D measuring laser microscopy images of EUF (Estelite Universal Flow). Bar scale = 50 µm.

**Figure 3:**
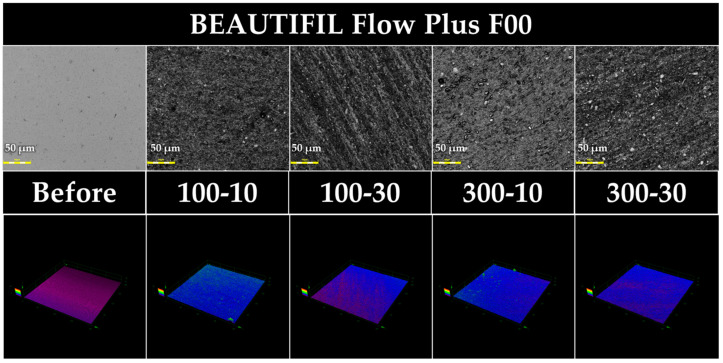
Representative 3D measuring laser microscopy images of BFP (BEAUTIFIL Flow Plus F00). Bar scale = 50 µm.

**Figure 4:**
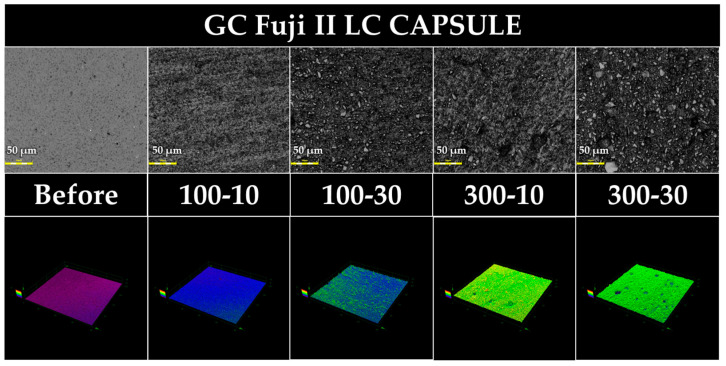
Representative 3D measuring laser microscopy images of FLC (GC Fuji II LC CAPSULE). Bar scale = 50 µm.

**Figure 5:**
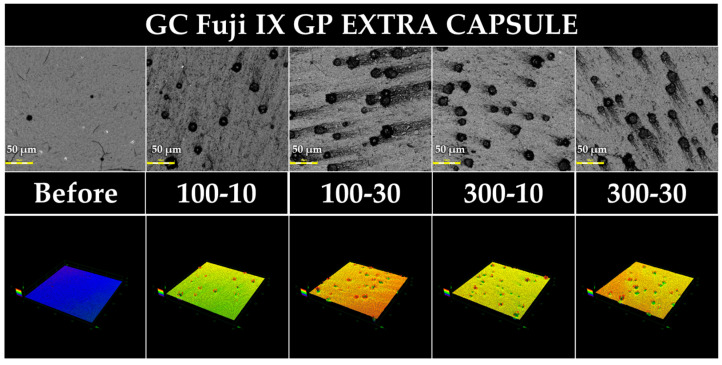
Representative 3D measuring laser microscopy images of FGP (GC Fuji IX GP EXTRA CAPSULE). Bar scale = 50 µm.

Figure 2, Figure 3, Figure 4 and Figure 5 show 3D measuring laser microscope images of the surface of a sample under the baseline and prophylaxis conditions. The upper and lower panels show images in 2D and 3D observation mode, respectively. In BFP and FLC, surface roughness was observed after prophylaxis compared with the findings at the baseline, and multiple craters were observed in FGP, suggesting that the glass powder had fallen out because of prophylaxis.

### 3.8. Scanning Electron Microscopy of Prophylaxis Paste Particles

**Figure 6:**
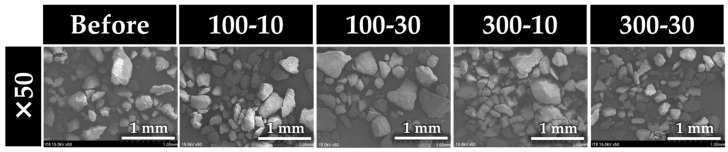
Representative SEM images of Prophy Paste Pro before (baseline) and after prophylaxis.

Figure 6 shows SEM images of the polished particles of Prophy Paste Pro collected after prophylaxis on the surface of EUF. After prophylaxis, the abrasive particles were finer than those of the baseline.

## 4. Discussion

To the authors’ knowledge, there are no clear guidelines for prophylaxis loading or the time per surface in dental practice. Christensen et al. [37] reported that 29 clinicians performed routine oral prophylaxes on 76 patients with an average micromotor speed of 2571 rpm, time per tooth surface of 4.5 s, and load of 145.4 gf. Hodges [38] also reported that a full-mouth prophylaxis takes 10 min. For 28 teeth, there are 76 tooth surfaces (12 labial and lingual surfaces of the premolars; 16 buccal, ligual, and occlusal surfaces of the molars; and 4 centric surfaces of the last molars). Therefore, the time spent per tooth surface is approximately 7.9 s. Onoue et al. [39] reported that the average daily prophylaxis load of five dental hygienists with more than 10 y of clinical experience was 331 gf. In this study, the rotational speed of the handpiece was set to 2500 rpm and a comparison was made between 100 and 300 gf, based on these reports. In this study, a one-step-type paste was used, in which abrasive particles were broken into smaller particles during use, and a slightly longer time setting was used than in previous reports [33].

In this study, the gloss value and three roughness parameters (Ra, Rz, Ry; widely used to evaluate the surface properties of restorative materials before and after polishing) were investigated. Rz (mean peak-to-valley height) can be calculated from the peak-to-valley values of five equal lengths. Ry (maximum roughness) is the distance between peak and valley points of the profile, which can be used as an indicator of the maximum defect height within the assessed profile. The effect of prophylaxis on the surface properties of the restorative materials was also evaluated by observing the microscopic 3D shape of the restorative material surface with a 3D laser microscope.

In preparing the baseline samples, those with bubbles, wrinkles, or scratches that were visually apparent were excluded. As a result, 40 specimens from each group were randomly assigned to four groups, and no significant differences were observed in the loss value or Ra value among the four groups. These results confirm that the random assignment resulted in an almost equal distribution of the samples. Encapsulated GICs have been reported to exhibit less variation in terms of the powder/liquid ratio than manually mixed GICs, with less variation depending on the skill of the operator [40,41,42]. It has also been reported that encapsulated GICs have higher mechanical properties because of less air bubble contamination [43]. In this study, the mechanical kneading types FLC and FGP were used with the aim of minimizing sample-to-sample variation.

The gloss and surface roughness of the two RBCs were similar. The filler loading of EUF (71 wt%, 57 vol%) is slightly higher than that of BFP (67.3 wt%, 47 vol%) [44]. Jassé et al. [45] fabricated RBCs with filler loadings of 40%, 50%, 60%, 70%, and 75%, and measured the gloss of the surface after pressure welding with a mylar strip. The gloss was highest at 75% and lowest at 40%. Therefore, filler loading might affect gloss and surface roughness. Similarly, Campbell et al. [46] observed that optical scattering by composite fillers linearly corresponded to the concentration of the filler material within the range of concentrations investigated. Therefore, not only filler loading but also several other factors such as filler particle size, shape, refractive index, and matrix resin properties correspond to the gloss as well as surface roughness of baseline samples [47].

At the baseline, the conventional GIC FGP was rougher and less reflective than two RBCs. In addition, the smoothness and gloss of the resin-modified GIC FLC were between that of the RBCs. Our results are similar to those in the literature [48,49]. In the 3D laser microscopy images, as in Komalsingsakul et al. [49], the EUF and BFP baseline images exhibited numerous small dot-like structures that appeared to be filler, but little waviness, a few scratches, and a few bubbles were observed, whereas the FLC image exhibited structures that appeared to be glass particles of various sizes and that were slightly convex. However, structures that appeared to be large and small glass particles were observed in FLC, and some unevenness was also observed. This might be the reason why not only Ra but also Rz and Ry were larger than those of the RBC. The Ry value of FGP was more than twice that of FLC. This is presumably because of microcracks on the surface of the specimens and some vacuoles that appeared to be shedding glass particles. Thus, even with careful surface polishing of the GIC, the immediate esthetic quality of the restoration is poorer than RBCs. This tendency was more pronounced with the FGP than FLC.

The surface roughness of EUF after prophylaxis did not change significantly in any of the four prophylaxis groups. However, all four prophylaxis groups exhibited a significant decrease in gloss, similar to the results reported by Sugiyama et al. [32]; i.e., the surface roughness of the RBC with a supra-nano spherical filler similar to EUF (Estelite Block, Tokuyama Dental) was significantly reduced. Regarding the CAD/CAM block (Estelite Block, Tokuyama Dental) with a supra-nano spherical filler similar to EUF, the gloss value decreased after prophylaxis even though the Ra value after prophylaxis was almost the same as that of the baseline. It is generally believed that the gloss value decreases with increasing roughness. However, it was reported that there is not always a negative correlation between the gloss and Ra values [27]. The 3D laser microscopy images exhibited no significant difference in roughness between the baseline and four prophylaxis groups, whereas the four prophylaxis groups in the 2D images exhibited no significant difference in the bathymetry of the baselines. In the 2D images, the four prophylaxis groups exhibited fine scratches that were not observed in the baseline. Therefore, the numerous shallow scratches, which do not affect the surface roughness, affected the light reflection and contributed to the reduction in the gloss level.

The gloss of BFP in the baseline was similar to that of EUF, but the decrease in gloss after prophylaxis was larger than that of EUF. Kameyama et al. [22] reported that the baseline gloss and Ra values of Clearfil AP-X (Kuraray Noritake Dental, Tokyo, Japan; containing irregular-shaped fillers with an average particle size of 3 µm) were not significantly different from those of Estelite Σ Quick (Tokuyama Dental; containing submicron spherical fillers with an average filler particle size of 0.4 µm). However, they also reported that the post-polishing gloss of Estelite Σ Quick was significantly higher than that of Clearfil AP-X [22]. Fillers contained in EUF consisted of supra-nano spherical filler with an average particle size of 0.2 µm. They are even smaller than Estelite Σ Quick. However, the average filler particle size of BFP is 0.8 µm (range of 0.1–4.0 µm) and it is irregularly shaped [50]. Hosoya et al. [51] compared the surface roughness of Beautifil II (Shofu) and Estelite Σ Quick, which used the same surface reaction pre-reacted glass–ionomer (S-PRG) filler as BFP, after grinding the surfaces with 180-, 1000-, and 3000-grit SiC paper. Beautifil II exhibited significantly larger Ra values in all cases. Therefore, the larger Ra value of BFP than that of EUF was assumed to be closely related to the shape and size of the filler in both cases.

Both EUF and BFP exhibited the largest decrease in gloss at 300 gf–30 s. Therefore, the fourth hypothesis (duration and load of dental prophylaxis does not affect surface gloss, roughness, and color change) was rejected. Shinkai et al. [52] reported that BFP exhibited greater wear in a three-body wear test, but comparatively less wear in a two-body wear test. Miyano et al. [50] compared the brushing wear characteristics of eight Beautifil Flow (Shofu) samples with different mean filler particle sizes and viscosities; BFP exhibited a certain degree of wear, and filler shedding was observed after brushing wear. The increase in average Ra and Ry values was larger than that of EUF, which was assumed to be caused by filler loss. Hosoya et al. [53] found that the surfaces of BFPs polished with 3000-grit SiC paper (with silicon carbide particles averaging 5 µm in diameter) exhibited Ra values averaging 0.5–1.0 µm and Gs values averaging 54.9–59.5%. The surfaces polished with a polishing disc for resin-based composites (Super-Snap and Shofu; with alumina particles of an average size of 7 µm) exhibited Ra values of an average of 1.3–1.4 µm and Gs values of ca. 10%. It is assumed that the surface of the EUF did not wear away after prophylaxis or that the filler as well as resin matrix wore away uniformly. However, the Ry value of BFP was significantly lower than that of EUF, but increased after prophylaxis, and this tendency was stronger as the load as well as time of prophylaxis increased. Thus, prophylaxis to BFP not only corresponded to a large difference in the degree of polishing of the filler and resin matrix, but also to the fact that the filler tended to drop out. Therefore, performing prophylaxis to BFPs in dental practice should be over a brief time under a small load.

FLC also exhibited a significant decrease in Gs after prophylaxis. The condition 100 gf–30 s exhibited more distinct surface irregularities than 100 gf–10 s in the 3D images obtained with a 3D measuring laser microscope. Thus, the matrix component around the core is more easily worn than the fluoroaluminosilicate glass that remains as the core after the acid–base reaction, resulting in formation of fine irregularities by prophylaxis. Some craters were observed at 300 gf, suggesting that the prophylaxis at higher loads caused the glass core to drop out. Therefore, as with BFP, when performing prophylaxis on FLCs in dental practice, it is desirable to perform prophylaxis over a brief time under a small load.

Unlike the other three materials, FGP exhibited no statistical difference in the gloss value between 100 gf–10 s and 300 gf–10 s. Surface roughness was decreased after dental prophylaxis. Therefore, the first, second, and third null hypotheses were partially accepted. The surface of FGP after prophylaxis was observed with a 3D measuring laser microscope, and numerous crater-like images were observed. The diameter of these crater-like images was approximately 10–20 µm, which is almost the same as the mean particle size of powder contained in FGP [49,54]. Therefore, fluoroaluminosilicate glass, a powder component of FGP, might have been lost by prophylaxis. The Ra, Rz, and Ry values of FGP after prophylaxis were rather small compared with those at the baseline; furthermore, the causal relationship between the 3D measuring laser microscope results and the surface roughness obtained with the surface profilometer is unclear. Therefore, further detailed investigation is needed.

A mean roughness of 0.2 μm is the critical threshold for bacterial plaque retention [55,56]. Moreover, roughness is a determining factor for staining [57]. The Ra of FGP was >0.2 µm even on smooth surfaces that were pressure-welded with mylar strips. Thus, FGP is problematic in terms of plaque retention. The mean Ra values of the other materials were also increased by prophylaxis, although most of them were not significantly different. Although this study simulated the surface properties at 1 y after prophylaxis visits every 3 mo (4×/y), acid exposure and daily self-care are also factors that can change surface roughness. Certainly, FLC and FGP can be expected to release fluoride ions and inhibit demineralization [58,59,60]. However, several recent studies indicate that the concentration of fluoride ions released from these materials is insufficient [49,61]. Komalsingsakul et al. also found that brushing on FLC and FGP increased surface roughness, and that brushing significantly increased the biovolume of *S. mutans*, rendering it substantially larger than on RBCs [49]. Therefore, secondary caries’ inhibitory effect of fluoride ions is unlikely to be excessive when restoring root surface caries in the GICs of patients who are elderly.

The obtained mean ΔE*ab values in each group were smaller than the threshold value (ΔE*ab > 3.3) at which humans can visually recognize color differences [62,63,64]. Although high water absorption and increased surface roughness of materials affect the discoloration of materials [65,66], the effect of prophylaxis on the color change was small.

This study simulates in vitro changes in restorative surface properties at 1 y after restoration with appropriate maintenance every 3 mo. However, in the actual oral cavity, self-care by brushing with toothpaste will change the surface properties [67]. Therefore, the actual intraoral environment will show further changes in surface properties. Further studies are needed to determine how more complex factors affect surface properties.

## 5. Conclusions

Based on the results of this study, the following conclusions are clarified:

1. Dental prophylaxis to direct dental restorative materials significantly reduces their surface gloss, except for FGP.
2. Dental prophylaxis to direct dental restorative materials significantly increases their surface roughness for EUF, BFP, and FLC. However, the surface roughness parameters of FGP are substantially decreased after dental prophylaxis.
3. Dental prophylaxis to direct dental restorative materials does not affect their color change.
4. The higher loadings and longer durations of dental prophylaxis might affect the surface gloss and roughness, depending on the material.

## Figures and Tables

**Table 1:** Materials used in this study.

Product Name (Manufacturer)	Shade	Principal Ingredient	Lot No.	Code
(Resin-based composite restorative material)				
Estelite Universal Flow (Super Low Flow) (Tokuyama Dental, Tokyo, Japan)	A3	Silica–zirconia filler, Bis-GMA, Bis-MPEPP, TEGDMA, UDMA, CQ, other	070021	EUF
BEAUTIFIL Flow Plus F00 (Shofu, Kyoto, Japan)	A3	Bis-GMA, TEGDMA, glass powder, other	042169	BFP
(Glass–ionomer-based restorative material)				
GC Fuji II LC CAPSULE (GC, Tokyo, Japan)	A3	Powder: fluoroaluminosilicate glass Liquids: methacrylic acid ester, polyacrylic acid, distilled water	2101121 2106122	FLC
GC Fuji IX GP EXTRA CAPSULE (GC, Tokyo, Japan)	A3	Powder: fluoroaluminosilicate glass, polyacrylic acid Liquids: polyacrylic acid, distilled water, polybasic carboxylic acid	2101252 2109201	FGP
(Prophylactic paste)				
Prophy Paste PRO (Directa AB, Upplands Väsby, Sweden)		Glycerin, sodium dihydrogen phosphate, water, other	33248	

Notes: Bis-GMA, bisphenol-glycidylmethacrylate; Bis-MPEPP, 2, 2′-bis (4-methacryloxy polyethoxyphenyl) propane; TEGDMA, triethylene glycol dimethacrylate; UDMA, urethane dimethacrylate; CQ, _DL_-camphorquinone.

**Table 2:** Differences in surface gloss (Gs) and roughness (Ra, Rz, Ry) measured at baseline.

	Gs (%)	Ra (µm)	Rz (µm)	Ry (µm)
Estelite Universal Flow	85.5 (5.8) ^a^	0.063 (0.013) ^a^	0.357 (0.108) ^a^	0.436 (0.128) ^b^
BEAUTIFIL Flow Plus	89.4 (2.9) ^a^	0.059 (0.013) ^a^	0.251 (0.088) ^a^	0.284 (0.102) ^a^
GC Fuji II LC CAPSULE	72.4 (6.3) ^b^	0.145 (0.023) ^b^	0.624 (0.130) ^b^	0.911 (0.177) ^c^
GC Fuji IX GP EXTRA CAPSULE	63.5 (9.9) ^c^	0.287 (0.061) ^c^	1.439 (0.354) ^c^	2.014 (0.441) ^d^

Mean (S.D.), *n* = 40. The same superscript letters indicate no statistically significant differences in the same column (*p* < 0.05).

**Table 3:** Differences in surface gloss (Gs) measured before and after prophylaxis.

	Baseline	After Prophylaxis	Δ*_Gs_*
Estelite Universal Flow			
100 gf–10 s	88.2 (4.4) ^a^	63.7 (5.1) ^def^	24.51
100 gf–30 s	82.4 (5.7) ^abc^	52.1 (4.7) ^ghi^	30.36
300 gf–10 s	84.2 (6.6) ^ab^	60.7 (6.8) ^efg^	23.52
300 gf–30 s	87.1 (5.4) ^a^	43.1 (3.3) ^ij^	43.96
BEAUTIFIL Flow Plus			
100 gf–10 s	88.1 (3.7) ^a^	56.6 (6.2) ^fgh^	31.48
100 gf–30 s	90.1 (2.7) ^a^	43.0 (11.4) ^ij^	47.09
300 gf–10 s	90.6 (2.1) ^a^	23.6 (5.3) ^kl^	67.00
300 gf–30 s	88.7 (2.4) ^a^	23.7 (11.6) ^kl^	64.95
GC Fuji II LC CAPSULE			
100 gf–10 s	71.5 (6.0) ^cde^	50.6 (6.0) ^ghi^	20.95
100 gf–30 s	72.0 (6.9) ^cde^	34.0 (4.0) ^jk^	38.00
300 gf–10 s	74.9 (5.4) ^bcd^	26.2 (6.1) ^kl^	48.72
300 gf–30 s	71.2 (7.0) ^cde^	15.1 (2.9) ^l^	56.13
GC Fuji IX GP EXTRA CAPSULE			
100 gf–10 s	61.6 (7.5) ^efg^	61.6 (4.3) ^efg^	−0.07
100 gf–30 s	61.3 (13.4) ^efg^	48.8 (7.9) ^hi^	12.51
300 gf–10 s	61.9 (10.0) ^efg^	55.8 (4.3) ^fgh^	6.19
300 gf–30 s	69.1 (6.4) ^de^	46.7 (9.5) ^hi^	22.39

Mean (S.D.), %, *n* = 10. The same superscript letters indicate no statistically significant differences in the same column (*p* < 0.05).

**Table 4:** Differences in surface roughness (Ra) measured before and after prophylaxis.

	Baseline	After Prophylaxis	Δ*_Ra_*
Estelite Universal Flow			
100 gf–10 s	0.064 (0.007) ^i^	0.058 (0.019) ^i^	−0.006
100 gf–30 s	0.068 (0.014) ^i^	0.070 (0.015) ^i^	0.002
300 gf–10 s	0.064 (0.017) ^i^	0.063 (0.014) ^i^	0.000
300 gf–30 s	0.057 (0.013) ^i^	0.069 (0.022) ^i^	0.012
BEAUTIFIL Flow Plus			
100 gf–10 s	0.050 (0.010) ^i^	0.059 (0.008) ^i^	0.009
100 gf–30 s	0.056 (0.010) ^i^	0.095 (0.026) ^hi^	0.039
300 gf–10 s	0.063 (0.013) ^i^	0.111 (0.020) ^ghi^	0.048
300 gf–30 s	0.066 (0.014) ^i^	0.135 (0.019) ^fgh^	0.069
GC Fuji II LC CAPSULE			
100 gf–10 s	0.151 (0.027) ^fgh^	0.157 (0.032) ^efgh^	0.006
100 gf–30 s	0.150 (0.024) ^fgh^	0.160 (0.060) ^efg^	0.010
300 gf–10 s	0.137 (0.021) ^fgh^	0.165 (0.040) ^defg^	0.029
300 gf–30 s	0.144 (0.021) ^fgh^	0.171 (0.029) ^defg^	0.028
GC Fuji IX GP EXTRA CAPSULE			
100 gf–10 s	0.271 (0.053) ^abc^	0.222 (0.045) ^bcd^	−0.049
100 gf–30 s	0.280 (0.080) ^ab^	0.217 (0.083) ^cde^	−0.063
300 gf–10 s	0.295 (0.056) ^a^	0.182 (0.054) ^def^	−0.114
300 gf–30 s	0.300 (0.056) ^a^	0.167 (0.037) ^defg^	−0.133

Mean (S.D.), μm, *n* = 10. The same superscript letters indicate no statistically significant differences in the same column (*p* < 0.05).

**Table 5:** Differences in surface roughness (Rz) measured before and after prophylaxis.

	Baseline	After Prophylaxis	*Δ_Rz_*
Estelite Universal Flow			
100 gf–10 s	0.445 (0.099) ^fgh^	0.347 (0.143) ^gh^	−0.098
100 gf–30 s	0.329 (0.085) ^gh^	0.305 (0.087) ^gh^	−0.024
300 gf–10 s	0.354 (0.106) ^gh^	0.339 (0.163) ^gh^	−0.015
300 gf–30 s	0.301 (0.098) ^gh^	0.316 (0.099) ^gh^	0.014
BEAUTIFIL Flow Plus			
100 gf–10 s	0.235 (0.092) ^h^	0.287 (0.092) ^gh^	0.052
100 gf–30 s	0.227 (0.062) ^h^	0.452 (0.099) ^fgh^	0.225
300 gf–10 s	0.270 (0.109) ^gh^	0.477 (0.095) ^fgh^	0.206
300 gf–30 s	0.273 (0.086) ^gh^	0.603 (0.092) ^efgh^	0.330
GC Fuji II LC CAPSULE			
100 gf–10 s	0.661 (0.111) ^efg^	0.806 (0.215) ^def^	0.145
100 gf–30 s	0.639 (0.150) ^efg^	0.874 (0.442) ^cde^	0.235
300 gf–10 s	0.586 (0.149) ^efgh^	0.940 (0.359) ^cde^	0.354
300 gf–30 s	0.611 (0.111) ^efgh^	0.775 (0.149) ^def^	0.163
GC Fuji IX GP EXTRA CAPSULE			
100 gf–10 s	1.339 (0.314) ^ab^	1.108 (0.263) ^bcd^	−0.231
100 gf–30 s	1.441 (0.447) ^ab^	1.237 (0.539) ^abc^	−0.205
300 gf–10 s	1.445 (0.279) ^ab^	0.899 (0.356) ^cde^	−0.547
300 gf–30 s	1.530 (0.383) ^a^	0.839 (0.253) ^def^	−0.691

Mean (S.D.), μm, *n* = 10. The same superscript letters indicate no statistically significant differences in the same column (*p* < 0.05).

**Table 6:** Differences in surface roughness (Ry) measured before and after prophylaxis.

	Baseline	After Prophylaxis	Δ*_Ry_*
Estelite Universal Flow			
100 gf–10 s	0.532 (0.123) ^hijk^	0.412 (0.160) ^jk^	−0.120
100 gf–30 s	0.422 (0.101) ^ijk^	0.383 (0.124) ^jk^	−0.039
300 gf–10 s	0.436 (0.124) ^ijk^	0.420 (0.184) ^jk^	−0.016
300 gf–30 s	0.355 (0.117) ^jk^	0.407 (0.152) ^jk^	0.052
BEAUTIFIL Flow Plus			
100 gf–10 s	0.278 (0.102) ^k^	0.360 (0.113) ^jk^	0.081
100 gf–30 s	0.260 (0.079) ^k^	0.623 (0.154) ^ghijk^	0.363
300 gf–10 s	0.312 (0.142) ^k^	0.687 (0.137) ^ghijk^	0.375
300 gf–30 s	0.284 (0.082) ^k^	0.831 (0.166) ^fghij^	0.546
GC Fuji II LC CAPSULE			
100 gf–10 s	0.969 (0.176) ^fgh^	1.096 (0.264) ^efg^	0.127
100 gf–30 s	0.951 (0.208) ^fgh^	1.209 (0.559) ^def^	0.258
300 gf–10 s	0.823 (0.176) ^fghij^	1.274 (0.343) ^def^	0.451
300 gf–30 s	0.900 (0.127) ^fghi^	1.172 (0.225) ^def^	0.272
GC Fuji IX GP EXTRA CAPSULE			
100 gf–10 s	1.871 (0.280) ^abc^	1.555 (0.357) ^cde^	−0.316
100 gf–30 s	1.927 (0.533) ^abc^	1.648 (0.646) ^bcd^	−0.278
300 gf–10 s	2.152 (0.458) ^a^	1.199 (0.355) ^def^	−0.954
300 gf–30 s	2.107 (0.456) ^ab^	1.205 (0.351) ^def^	−0.902

Mean (S.D.), μm, *n* = 10. The same superscript letters indicate no statistically significant differences in the same column (*p* < 0.05).

**Table 7:** Mean ΔE*ab values in each group.

	Estelite Universal Flow	Beautifil Flow Plus	GC Fuji II LC CAPSULE	GC Fuji IX GP EXTRA CAPSULE
100 gf–10 s	0.391	0.594	0.450	0.124
100 gf–30 s	0.185	2.100	0.995	0.200
300 gf–10 s	0.692	1.020	0.659	0.341
300 gf–30 s	0.516	1.021	1.673	0.224

*n* = 10.

## Data Availability

Data are available in this article.
